# Supplementary material for: Different Recognition of Protein Features Depending on Deep Learning Models: A Case Study of Aromatic Decarboxylase UbiD
Source: Biology (Basel). 2023 May 31;12(6):795. doi: 10.3390/biology12060795 (PMC10295083; doi:10.3390/biology12060795)
Supplement: Supplementary file 1 [file biology-12-00795-s001.zip › Supplemental information.pdf]

Supplementary information

Different recognition of protein features depending on deep learning models: a case study of aromatic decarboxylase UbiD

Naoki Watanabe†, Yuki Kuriya†, Masahiro Murata, Masaki Yamamoto, Masayuki Shimizu and Michihiro Araki*
†These authors contributed equally to the work.

Supplementary Figures

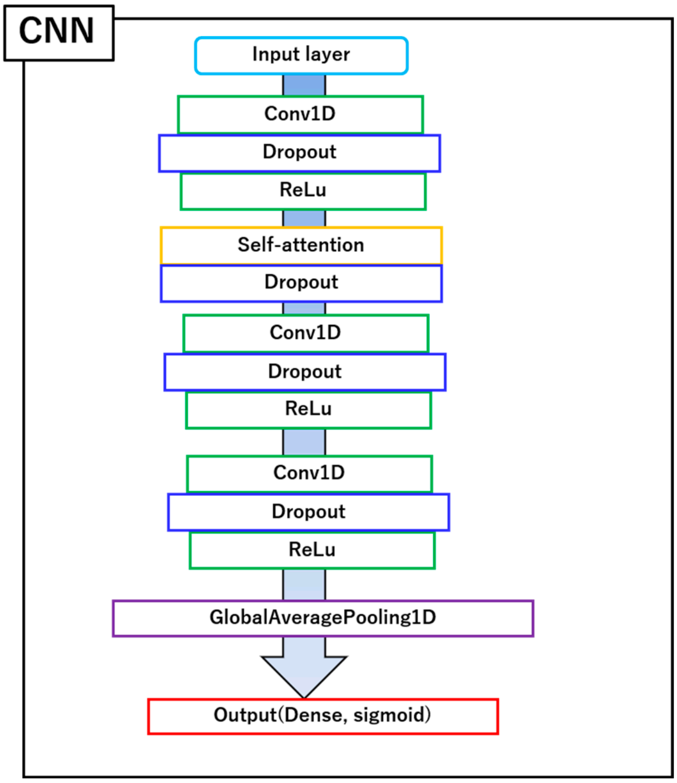

Supplementary Figure S1. CNN model architecture for predicting target enzymes.

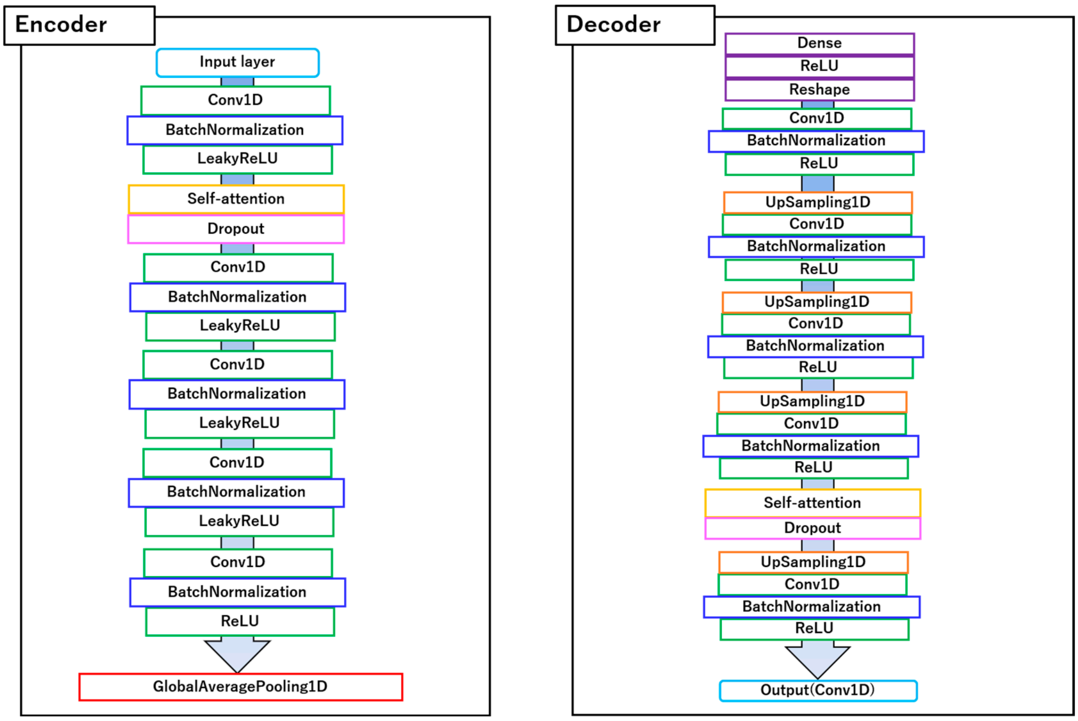

Supplementary Figure S2. CNN-AE model architecture for extracting target enzyme sequence features.

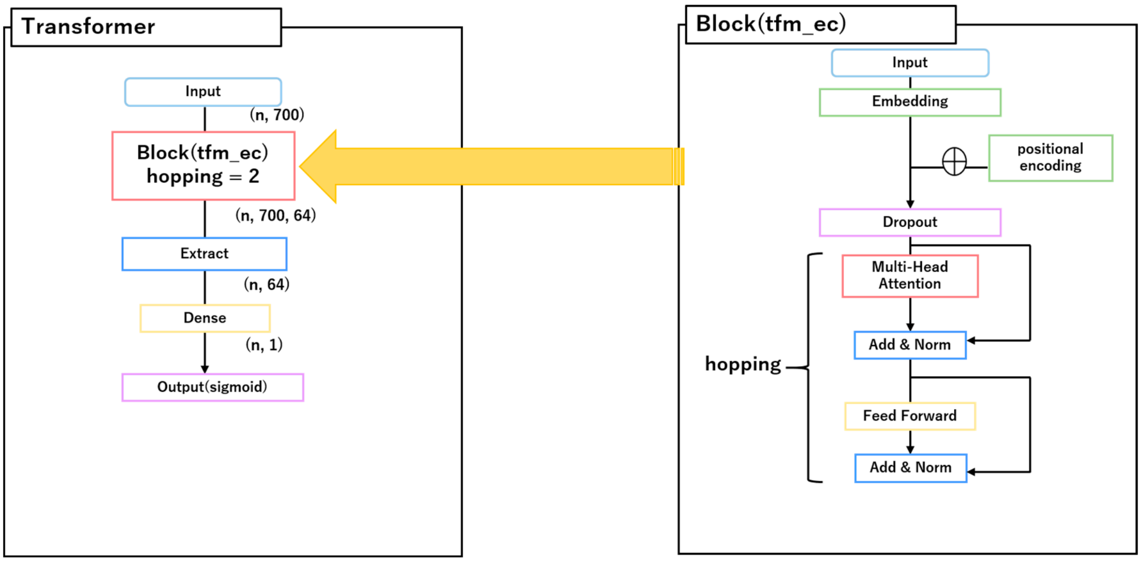

Supplementary Figure S3. Architecture of transformer model for predicting target enzymes and extracting target enzyme sequence features (left panel). The right panel shows Block (tfm_ec) architecture in the encoder of transformer model [1].

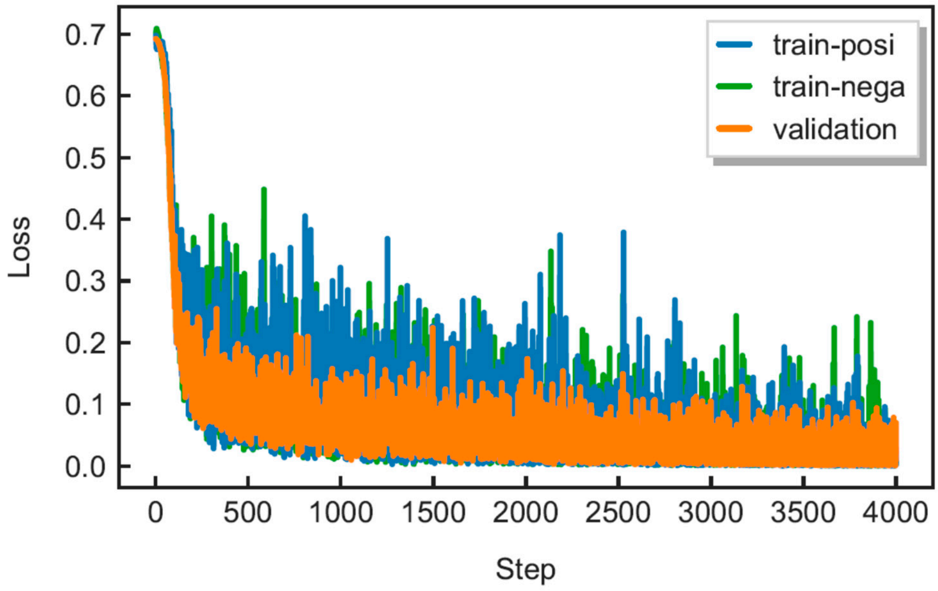

Supplementary Figure S4. Training for positive samples (blue line), training for negative samples (green line) and validation (orange line) loss curves of CNN model for 4,000 steps.

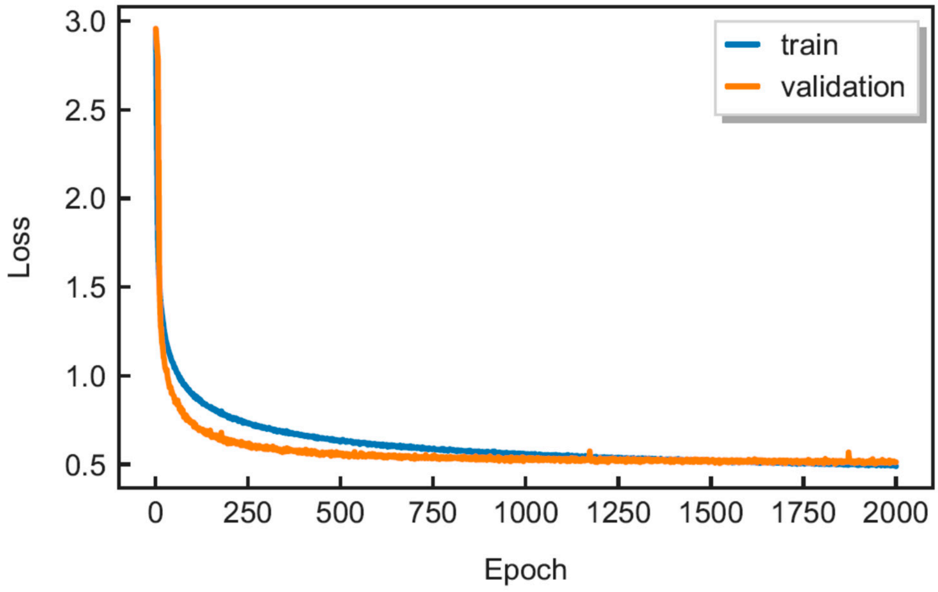

Supplementary Figure S5. Training (blue line) and validation (orange line) loss curves of CNN-AE model for 2,000 epochs.

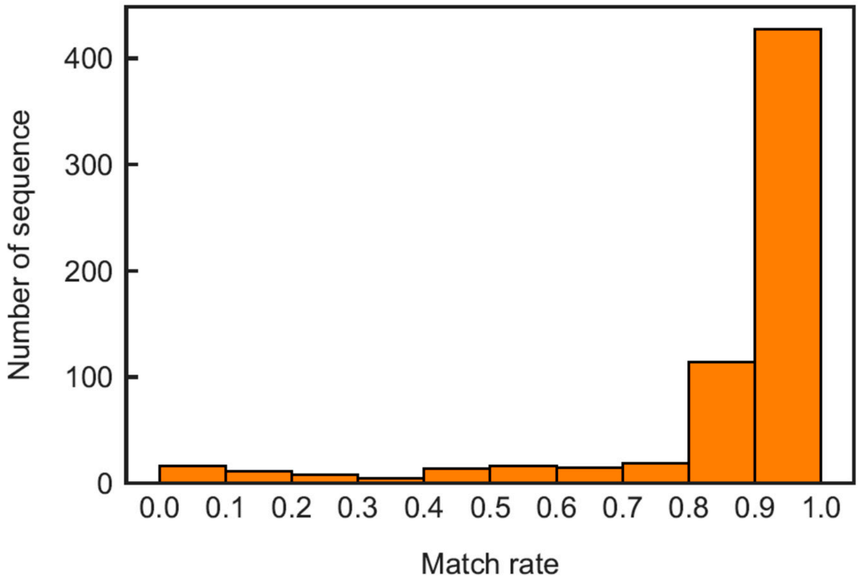

Supplementary Figure S6. Histogram of match rates between output sequences and input sequences using CNN-AE model in 2,000 epochs. The horizontal axis shows Match rate between the output sequences to the input sequences, while the vertical axis shows the number of the sequences.

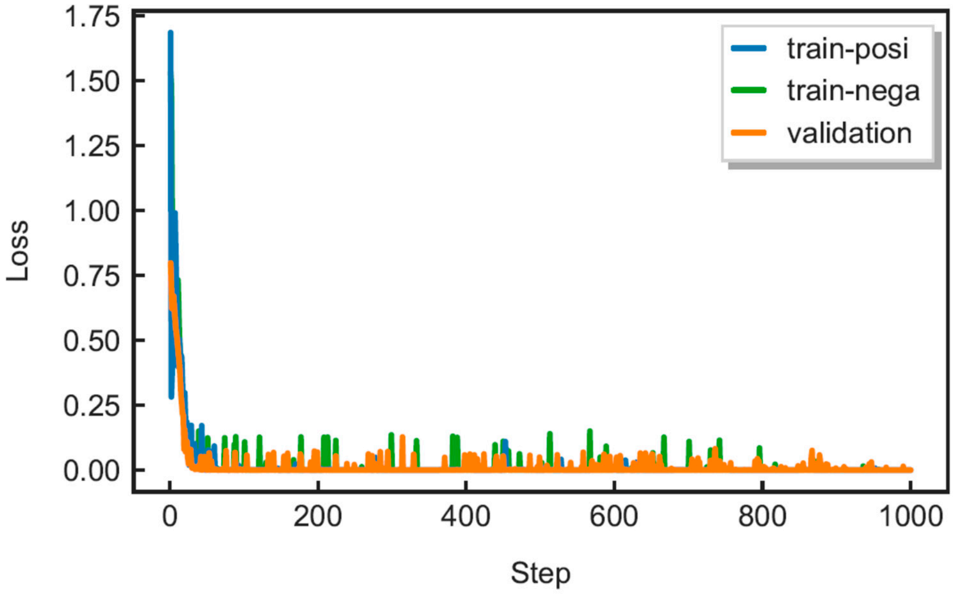

Supplementary Figure S7. Training for positive samples (blue line), training for negative samples (green line) and validation (orange line) loss curves of Transformer model for 1,000 steps.

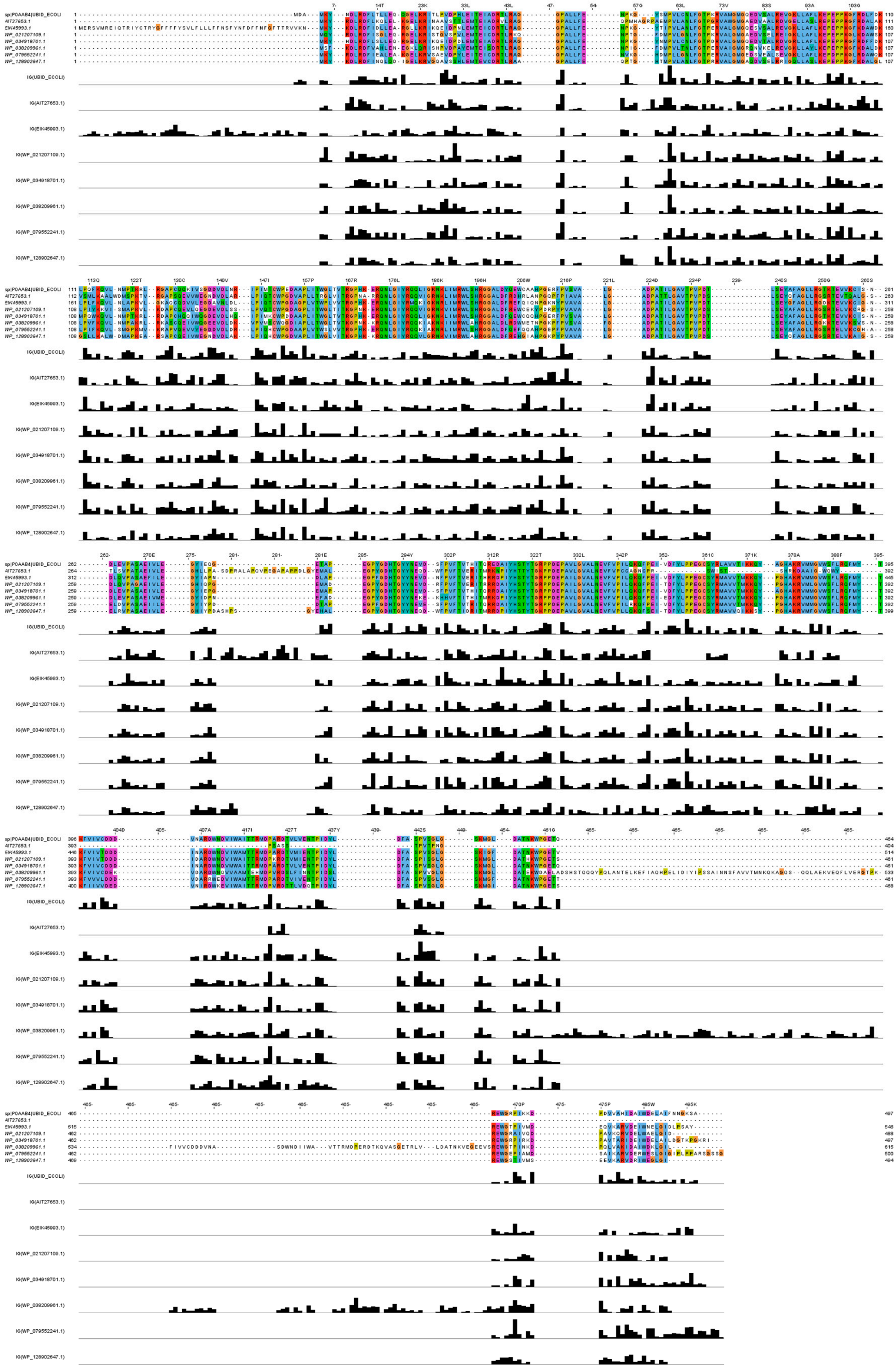
sp|P0AAB4|UBID_ECOLI
AIT27653.1
EIK45993.1
WP_021207109.1
WP_034918701.1
WP_038209961.1
WP_079552241.1
WP_128902647.1
IG(UBID_ECOLI)
IG(AIT27653.1)
IG(EIK45993.1)
IG(WP_021207109.1)
IG(WP_034918701.1)
IG(WP_038209961.1)
IG(WP_079552241.1)
IG(WP_128902647.1)

Supplementary Figure S8. Multiple sequence alignment for *Escherichia coli* UbiD (UBID_ECOLI) and representative UbiD sequences (Table S2) and IG results of representative UbiD sequences derived from classification scores using CNN model. Bar charts show the IG values which are normalized between 0 and 1.

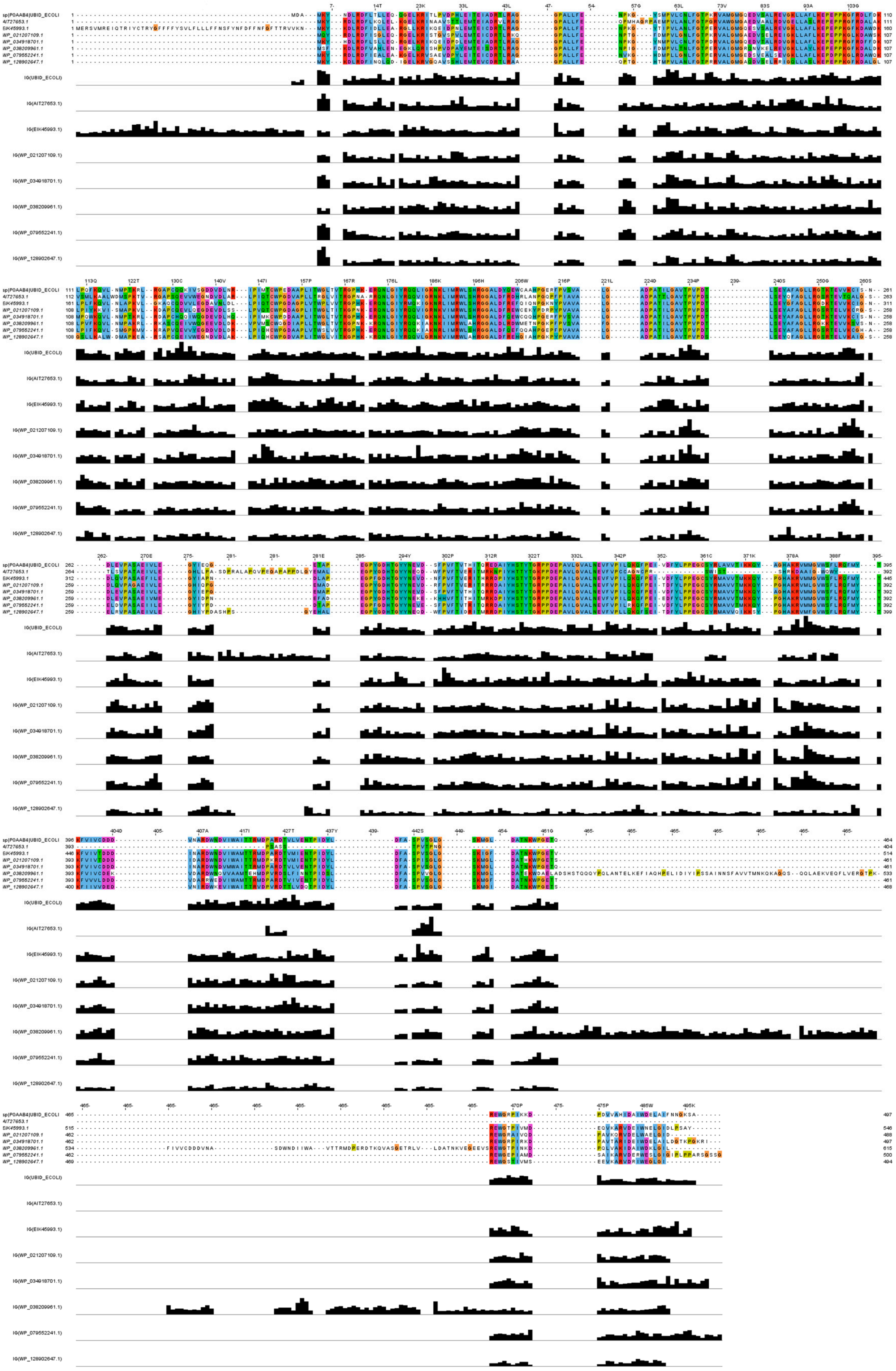
sp|P0AAB4|UBID_ECOLI
AIT27653.1
EIK45993.1
WP_021207109.1
WP_034918701.1
WP_038209961.1
WP_079552241.1
WP_128902647.1
IG(UBID_ECOLI)
IG(AIT27653.1)
IG(EIK45993.1)
IG(WP_021207109.1)
IG(WP_034918701.1)
IG(WP_038209961.1)
IG(WP_079552241.1)
IG(WP_128902647.1)

Supplementary Figure S9. Multiple sequence alignment for *E. coli* UbiD (UBID_ECOLI) and representative UbiD sequences (Table S2) and IG results of representative UbiD sequences derived from feature vectors using CNN-AE model. Bar charts show the IG values which are normalized between 0 and 1.

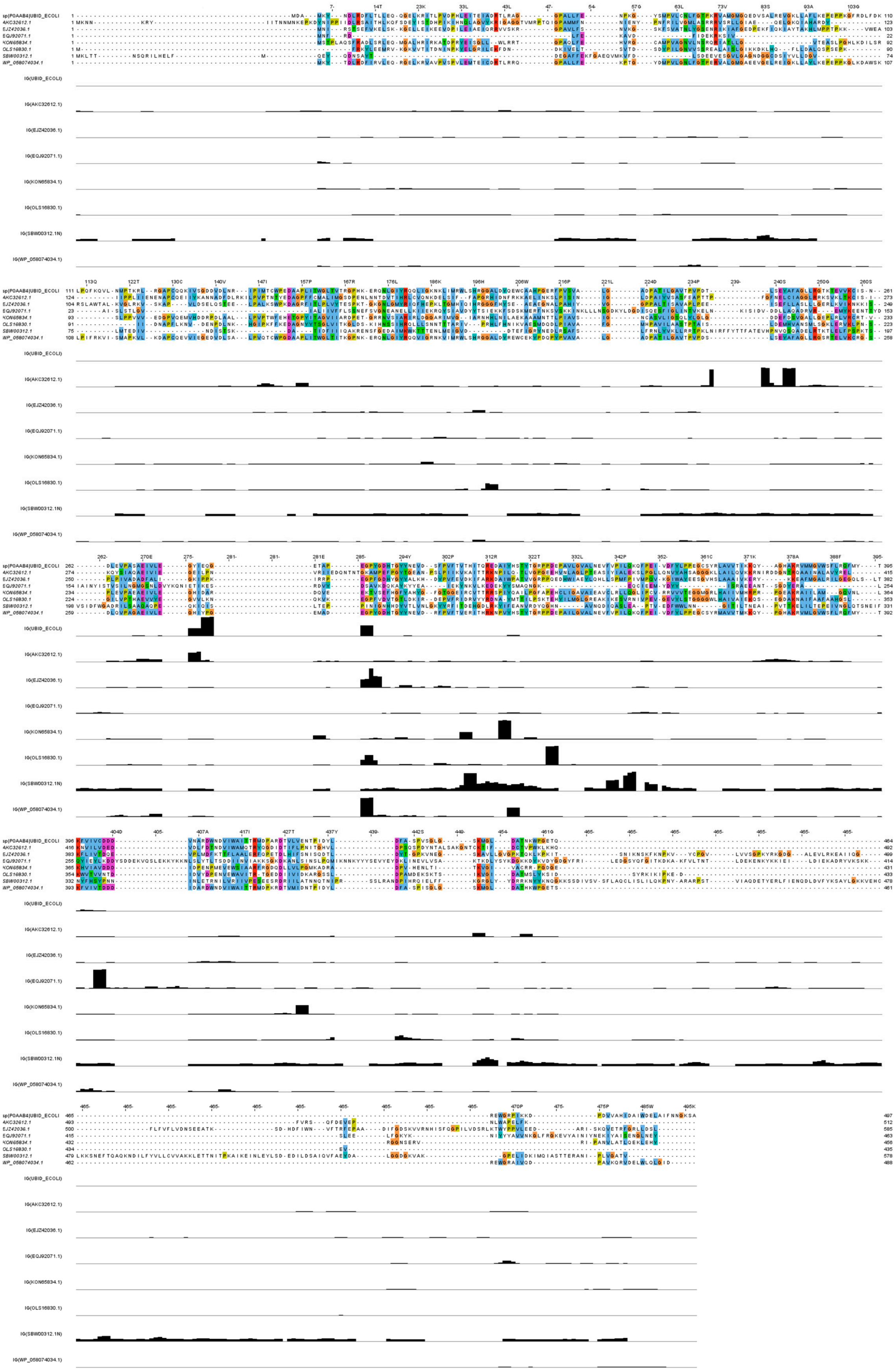
sp|P0AAB4|UBID_ECOLI
AKC32612.1
EJZ42036.1
EQJ92071.1
KON65834.1
OLS16830.1
SBW00312.1
WP_058074034.1
IG(UBID_ECOLI)
IG(AKC32612.1)
IG(EJZ42036.1)
IG(EQJ92071.1)
IG(KON65834.1)
IG(OLS16830.1)
IG(SBW00312.1N)
IG(WP_058074034.1)

Supplementary Figure S10. Multiple sequence alignment for *E. coli* UbiD (UBID_ECOLI) and representative UbiD sequences (Table S2) and IG results of representative UbiD sequences derived from feature vectors using Transformer model. Bar charts show the IG values which are normalized between 0 and 1.

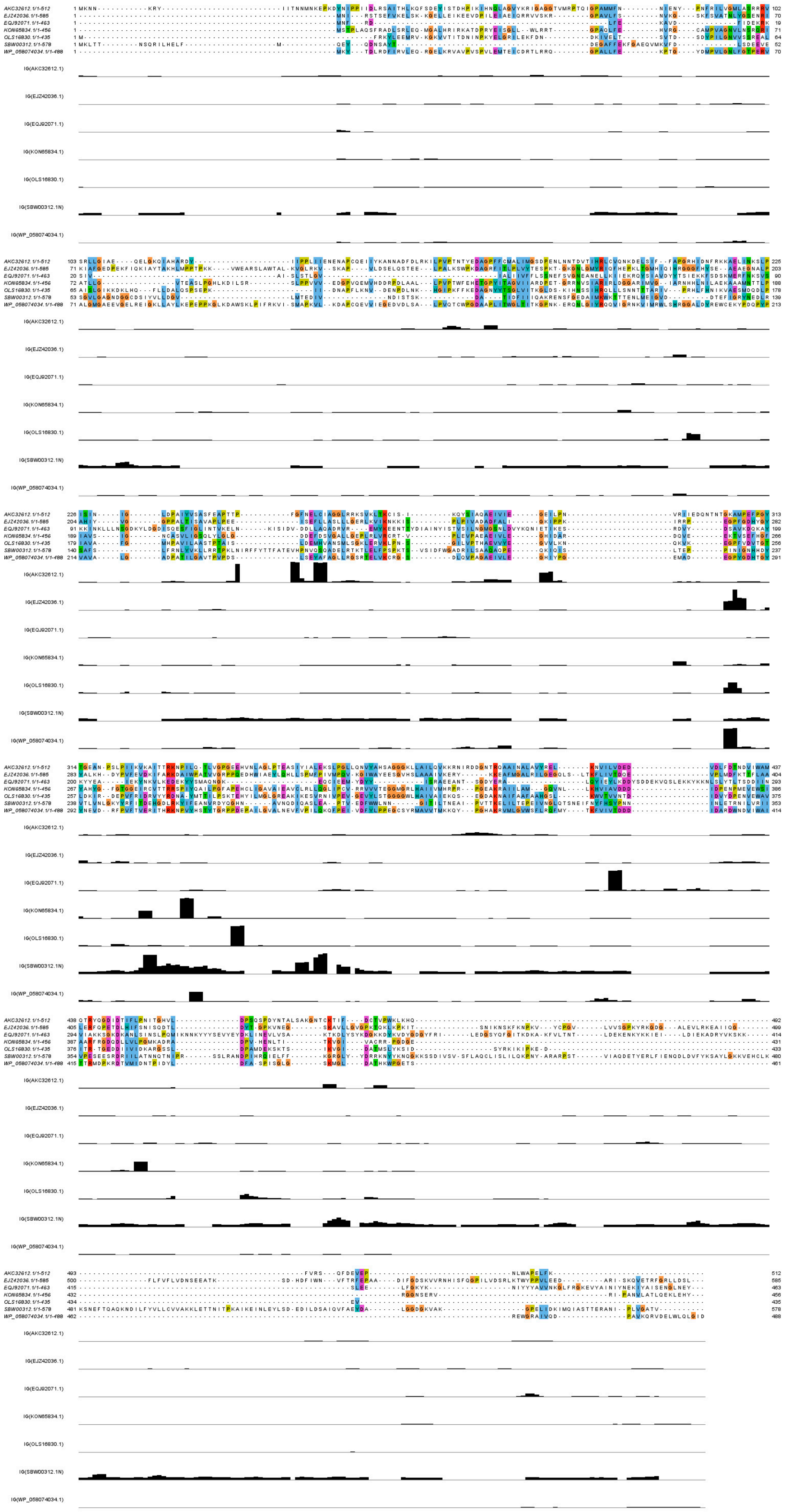
AKC32612.1/1-512
EJZ42036.1/1-585
EQJ92071.1/1-463
KON65834.1/1-456
OLS16830.1/1-435
SBW00312.1/1-578
WP_058074034.1/1-488
IG(AKC32612.1)
IG(EJZ42036.1)
IG(EQJ92071.1)
IG(KON65834.1)
IG(OLS16830.1)
IG(SBW00312.1N)
IG(WP_058074034.1)

Supplementary Figure S11. Multiple sequence alignment for *E. coli* UbiD (UBID_ECOLI) and representative UbiD sequences (Table S2) and IG results of representative UbiD sequences derived from classification scores using Transformer model. Bar charts show the IG values which are normalized between 0 and 1.

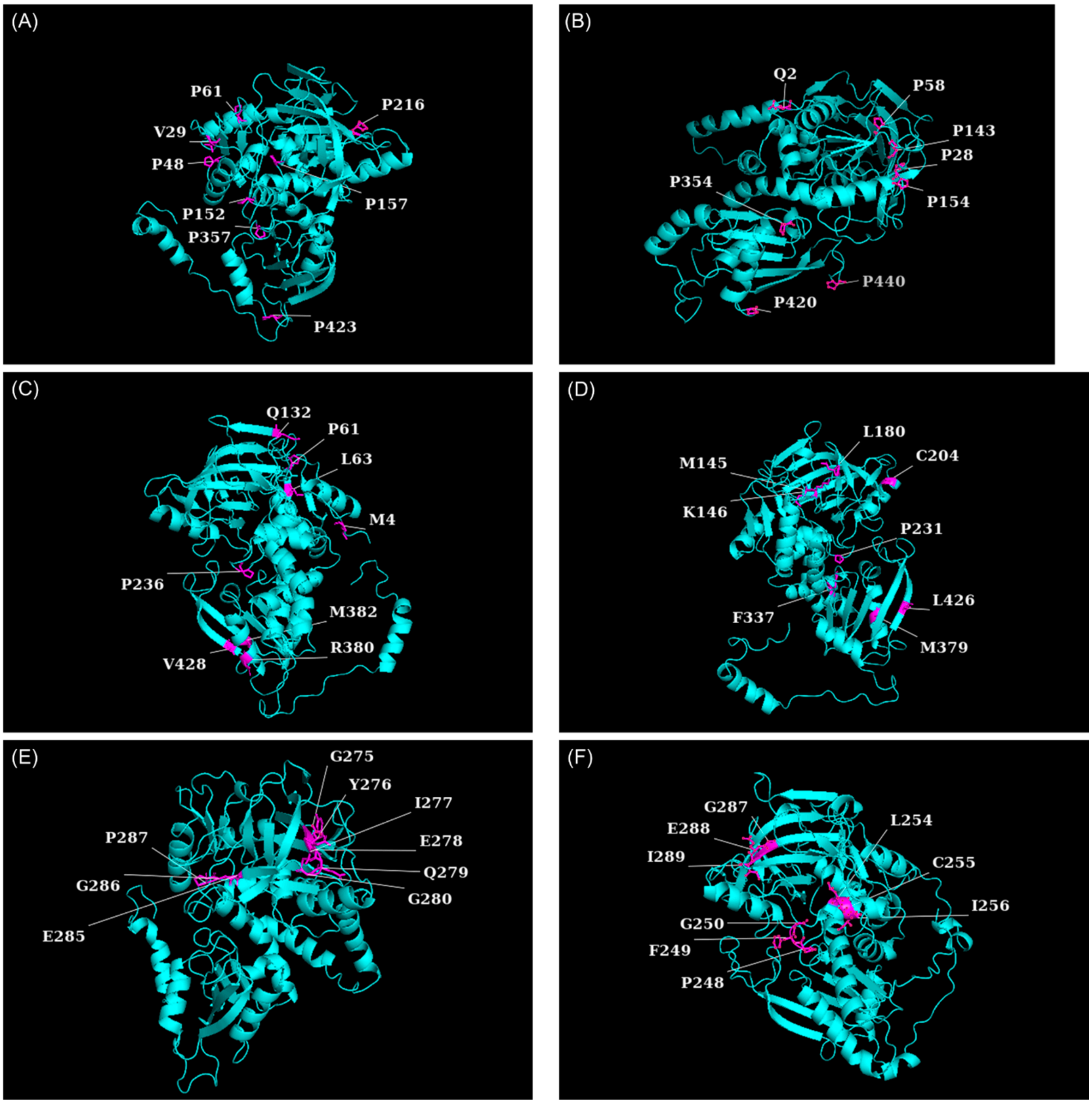

Supplementary Figure S12. UbiD structures using ESMFold [2] structure prediction and IG results. The structures were visualized by Pymol [3]. The resiudes with higher IG values are shown in magentas. (A) *E. coli* UbiD results using CNN model, (B) WP_021207109.1 results using CNN model, (C) *E. coli* UbiD results using CNN-AE model, (D) WP_034918701.1 results using CNN-AE model, (E) *E. coli* UbiD results using Transformer 1 model and (F) AKC32612.1 results using CNN-AE model. The

residue numbers of (B), (D) and (F) figures are of each sequence not *E. coli* UbiD.

Supplementary Table

Supplementary Table S1. Test evaluations for CNN and Transformer models.

CNN

Step No.	ACC	AUC	F_1 score	MCC
100	0.500	0.967	0.000	-0.008
500	0.942	0.991	0.917	0.911
1,000	0.963	0.994	0.935	0.930
1,500	0.969	0.995	0.954	0.950
2,000	0.978	0.996	0.968	0.965
2,500	0.986	0.996	0.975	0.973
3,000	0.976	0.997	0.970	0.968
3,500	0.992	0.997	0.975	0.974
4,000	0.993	0.997	0.982	0.981

Transformer

Step No.	ACC	AUC	F_1 score	MCC
100	0.997	0.999	0.994	0.993
200	0.996	0.999	0.992	0.992
300	0.998	0.999	0.995	0.995
400	0.998	0.999	0.996	0.996
500	0.998	0.999	0.997	0.997
600	0.998	0.998	0.997	0.997
700	0.998	0.998	0.996	0.996
800	0.997	0.998	0.996	0.996
900	0.997	0.999	0.997	0.997
1,000	0.998	0.999	0.998	0.997

Supplementary Table S2. Representative sequences selected from each cluster derived from clustering by feature vectors of CNN-AE and Transformer models.

CNN-AE

Accession No.	Strain	Annotation
AIT27653.1	*Bordetella holmesii 44057*	ubiD decarboxylase family protein
EIK45993.1	*Cellvibrio sp. BR*	3-octaprenyl-4-hydroxybenzoate carboxy-lyase
WP_021207109.1	*Pseudomonas stutzeri*	4-hydroxy-3-polyprenylbenzoate decarboxylase
WP_034918701.1	*Erwinia sp. 9145*	4-hydroxy-3-polyprenylbenzoate decarboxylase
WP_038209961.1	*Vibrio tubiashii*	4-hydroxy-3-polyprenylbenzoate decarboxylase
WP_079552241.1	*Halomonas subglaciescola*	4-hydroxy-3-polyprenylbenzoate decarboxylase
WP_128902647.1	*Janthinobacterium sp. 17J80-10*	4-hydroxy-3-polyprenylbenzoate decarboxylase

Transformer

Accession No.	Strain	Description
AKC32612.1	*Candidatus Pantoea carbekii*	3-octaprenyl-4-hydroxybenzoate carboxy-lyase
EJZ42036.1	*Leptospira licerasiae str. MMD4847*	UbiD family decarboxylase
EQJ92071.1	*Clostridioides difficile P50*	ubiD family decarboxylase
KON65834.1	*Komagataeibacter europaeus*	3-octaprenyl-4-hydroxybenzoate carboxy-lyase
OLS16830.1	*Candidatus Heimdallarchaeota archaeon LC_2*	3-octaprenyl-4-hydroxybenzoate carboxy-lyase
SBW00312.1	uncultured Clostridiales bacterium	UbiD family decarboxylase
WP_058074034.1	*Pseudomonas*	4-hydroxy-3-polyprenylbenzoate decarboxylase

Supplementary Table S3. Euclidean distances of feature vectors and bitscores using BLASTp, Basic Local Alignment Search Tool [4] between *E. coli* UbiD and each representative sequence derived from CNN-AE and Transformer models.

CNN-AE

Accession No.	Feature vector distance	BLASTp bitscore
AIT27653.1	2.879	459
EIK45993.1	2.914	808
WP_021207109.1	3.994	796
WP_034918701.1	4.003	931
WP_038209961.1	3.183	744
WP_079552241.1	4.103	783
WP_128902647.1	3.475	725

Transformer Accession No.	Feature vector distance	BLASTp bitscore
WP_058074034.1	0.72	792
KON65834.1	2.11	142
EQJ92071.1	3.95	22.3
EJZ42036.1	4.06	216
OLS16830.1	7.27	136
AKC32612.1	10.12	119
SBW00312.1	15.72	17.7

Supplementary Table S4. All euclidean distances of feature vectors between 2 sequences for representative sequences derived from (A) CNN-AE and (B) Transformer models.

CNN-AE	AIT27653.1	EIK45993.1	WP_021207109.1	WP_034918701.1	WP_038209961.1	WP_079552241.1	WP_128902647.1
AIT27653.1	0.000	1.864	4.360	4.424	3.006	4.164	3.146
EIK45993.1	1.864	0.000	4.661	4.652	2.978	4.271	3.242
WP_021207109.1	4.360	4.661	0.000	2.944	3.594	2.994	3.589
WP_034918701.1	4.424	4.652	2.944	0.000	3.646	3.216	3.705
WP_038209961.1	3.006	2.978	3.594	3.646	0.000	3.587	3.059
WP_079552241.1	4.164	4.271	2.994	3.216	3.587	0.000	3.399
WP_128902647.1	3.146	3.242	3.589	3.705	3.059	3.399	0.000

Transformer	AKC32612.1	EJZ42036.1	EQJ92071.1	KON65834.1	OLS16830.1	SBW00312.1	WP_058074034.1
AKC32612.1	0.000	10.826	8.155	9.518	8.995	12.242	10.130
EJZ42036.1	10.826	0.000	6.877	4.930	5.478	15.282	4.500
EQJ92071.1	8.155	6.877	0.000	3.170	8.306	15.196	3.702
KON65834.1	9.518	4.930	3.170	0.000	7.099	15.653	2.222
OLS16830.1	8.995	5.478	8.306	7.099	0.000	13.844	7.615
SBW00312.1	12.242	15.282	15.196	15.653	13.844	0.000	15.699
WP_058074034.1	10.130	4.500	3.702	2.222	7.615	15.699	0.000

Supplementary Table S5. The results of the correlations between IG scores for each model and sequence conservation of UbiD *E. coli* [5]. The values are average correlation coefficients of UbiD E. coli and representative UbiD sequences. The correlation coefficients are Spearman's rank correlation coefficient.

Model	Correlation coefficient	Model	Correlation coefficient
CNN	-0.088	CNN-AE	-0.038
Transformer 1	0.019	Transformer 2	0.012

Reference

[1] Vaswani, A.; Shazeer, N.; Parmar, N.; Uszkoreit, J.; Jones, L.; Gomez, A. N.; Kaiser, Ł.; Polosukhin, I. Attention Is All You Need. In Proceeding of the

Advances in Neural Information Processing Systems 30, California, USA, 4-9 December 2017.
[2] Lin Z, Akin H, Rao R, Hie B, Zhu Z, Lu W, et al. Evolutionary-scale prediction of atomic-level protein structure with a language model. *Science* **2023**, *379*, 1123–1130. https://doi.org/10.1126/science.ade2574.
[3] The PyMOL Molecular Graphics System, Version 2.0 Schrödinger, LLC. Available online: https://pymol.org/2/ (accessed on 15 May 2023)
[4] Camacho C, Coulouris G, Avagyan V, Ma N, Papadopoulos J, Bealer K, et al. BLAST+: architecture and applications. *BMC Bioinformatics* **2009**, *10*, 421. https://doi.org/10.1186/1471-2105-10-421.
[5] Jacewicz A, Izumi A, Brunner K, Schnell R, Schneider G. Structural Insights into the UbiD Protein Family from the Crystal Structure of PA0254 from Pseudomonas aeruginosa. *PLoS One* **2013**, *8*, 1-10. https://doi.org/10.1371/journal.pone.0063161.
